# Molecular assays to reliably detect and quantify predation on a forest pest in bats faeces

**DOI:** 10.1038/s41598-022-06195-7

**Published:** 2022-02-10

**Authors:** Unai Baroja, Inazio Garin, Nerea Vallejo, Amaia Caro, Carlos Ibáñez, Andrea Basso, Urtzi Goiti

**Affiliations:** 1 Department of Zoology and Animal Cell Biology, Faculty of Science, University of the Basque Country, UPV/EHU, Leioa, Basque Country Spain; 2 Department of Zoology and Animal Cell Biology, Faculty of Pharmacy, University of the Basque Country, UPV/EHU, Vitoria-Gasteiz, Basque Country Spain; 3 Department of Evolutionary Ecology, Estación Biológica de Doñana (CSIC), Avenida Américo Vespucio 26, 41092 Seville, Spain; 4 Istituto Zooprofilattico Sperimentale delle Venezie, Viale dell’Università, 10, 35020 Legnaro, Padova Italy

**Keywords:** Ecosystem services, Invasive species, Molecular ecology

## Abstract

Targeted molecular methods such as conventional PCR (cPCR) and quantitative PCR (qPCR), combined with species-specific primers and probes, are widely applied for pest species detection. Besides, the potential of qPCR to quantify DNA in samples makes it an invaluable molecular tool to infer the predation levels on specific prey by analysing predators’ stools. Nevertheless, studies on the diet of bats failed to find any empirical relationship, and it remains to be evaluated. Thus, we developed and evaluated two species-specific PCR assays to detect and quantify DNA of a major forest pest, the pine processionary, *Thaumetopoea pityocampa,* in bats’ faeces. Further, we empirically compared a range of different known DNA concentrations (input) of the target species mixed with mocks and bat faecal samples against DNA abundances yielded by qPCR (output) for a quantitative assessment. Overall, cPCR showed a lower detection rate than qPCR, but augmenting the replicate effort from one to three replicates led to a greater increase in the detection rate of the cPCR (from 57 to 80%) than the qPCR (from 90 to 99%). The quantitative experiment results showed a highly significant correlation between the input and output DNA concentrations (t = 10.84, p < 0.001) with a mean slope value of 1.05, indicating the accuracy of our qPCR assay to estimate DNA abundance of *T. pityocampa* in bat faeces. The framework of this study can be taken as a model to design similar assays applicable to other species of interest, such as agricultural pests or insects of public health concern.

## Introduction

The pine processionary moth, *Thaumetopoea pityocampa*, is a significant forest pest of coniferous trees in the Western Palearctic. The larvae not only defoliate trees causing significant growth reduction and leading to severe economic losses^1,2^, but its urticating setae are also considered a risk to human and animal health^3,4^. The species is rapidly responding to climate change prolonging its active period and spreading northward^5–7^. Among its natural predators, insectivorous bats are common predators of adult moths^8^, with a vast array of bat species preying on *T. pityocampa*^9^. These bat species increase their hunting activity at pine stand edges where *T. pityocampa* swarms^10^. Some of these bat species have a broad foraging range^11–13^, reaching prey-abundant distant areas. During the breeding period, female bats increase their food intake imposed by pregnancy’s high energetic demands^14^, which coincides with the high resource availability period (e.g., the outbreak of *T. pityocampa*). In summary, these flying mammals may precisely track the abundance of *T. pityocampa* and other species of interest (e.g.^15^).

Current molecular techniques for identifying bat prey in diet studies are primarily based on the metabarcoding of prey DNA in faeces (e.g.^16–18^). The high throughput sequencing of thousands of PCR amplicons in parallel renders this technique a powerful tool to study animals’ diets with a high diversity of consumed taxa^19,20^. Nonetheless, specialised laboratory equipment and personnel are required, and the budget needed to process the data makes it impractical in many research settings. Moreover, metabarcoding brings an imbalanced amplification of the prey’s DNA attributed to the differential affinity of the primer sets across taxa, which can potentially lead to false-negative results of some prey^21^. Likewise, as most insectivorous bats have diverse diets, the large number of DNA sequences from multiple taxa binding to universal primers can potentially disguise the detection of some species of interest^20,22,23^. In that sense, although it is tempting to assume that prey DNA sequence proportions or read counts recovered from faecal samples are representative of predator’s diet proportions, digestion and amplification biases impair quantitative interpretations of the data, especially in samples containing many taxa^24,25^. Consequently, reliable results of metabarcoding restrict to the qualitative assessment of the species list in the faecal samples. Hence, whereby a single-species detection is aimed, metabarcoding may fail to reliably capture the target species leading to biased results. It is therefore desirable that cheaper, targeted approaches are developed for studies which aim to detect a single species. The combination of target species-specific primers with PCR-based methods provides greater detection than metabarcoding because they only target the species of interest. Among targeted approaches, conventional PCR (cPCR) and real-time or quantitative PCR (qPCR) methods allow the amplification of minute amounts of template DNA even when the target is mixed with large amounts of non-target DNA, such as in animal scat^26^.

Work from several fields have succeeded in the direct detection of target species using targeted PCR methods from environmental samples, for instance, water^27,28^, stomach content^26^, predator and prey identification from its scats^29–31^, plant tissue^32^ or soil samples^33^. Particularly, when working with bat faeces, several factors directly influence target species’ detection, primarily the mass of prey consumed and faecal collection time post-feeding, which can lead to false-negative results^31^. In addition, choosing the proper PCR strategy may sometimes result in a hard striking decision since both assays have their benefits and drawbacks^34^. Although cPCR is one of the most widespread and affordable molecular techniques, it allows amplifying a target sequence only at a qualitative level. In contrast, qPCR monitors the amplification process in real-time, and hence it provides a qualitative and quantitative assessment of the aimed sequence, but at a much higher cost. Another determining factor is the number of replicates used. Increasing PCR replicates significantly improves the detection probability by reducing the risk of false negatives and yielding more reliable results^35^. Making all these decisions (choosing PCR strategy or the number of replicates included) will involve a trade-off between the financial costs, logistical feasibility, and the risk of inaccurate results.

Despite the potential of qPCR to quantify DNA in samples, results obtained from these analyses have not yet shown correlations with the biomass of prey consumed by bats^36,37^. Indeed, several factors, such as degradation of dietary DNA during digestion or primer binding biases attributed to highly diverse prey diets, might hinder the potential of qPCR as a quantitative measure of predation.

In light of the above, the main goals of this study were to (1) develop rapid, easy and cost-effective PCR-based methods for detecting *T. pityocampa* in bat stools, (2) to compare the detection rates yielded by both cPCR and qPCR strategies along with different replicates and (3) to assess the accuracy of qPCR as a tool for estimating the abundance of *T. pityocampa* consumed by insectivorous bats.

## Material and methods

### Primer design

For cPCR, we designed specific primer sets to *T. pityocampa* from the 3ʹ end of TRNK to the 5ʹ of ATP8 regions of the mitochondrial DNA using PerlPrimer software (Ref.^38^, see Table 1). For that, we first downloaded all the available sequences from their congeneric species (*Thaumetopoea* spp., see Ref.^39^) on GenBank (www.ncbi.nlm.nih.gov/genbank/) and BOLD (www.boldsystems.org). Then we aligned them using MAFFT^40^, and we identified binding sites. Finally, we verified primers’ specificity in silico against available sequence data in the GenBank nucleotide database (https://www.ncbi.nlm.nih.gov/genbank/). For qPCR, we designed *T. pityocampa* specific primers and Taq-man probes optimised for qPCR reactions using the PrimerQuest Design Tool (Integrated DNA Technologies, https://eu.idtdna.com/) for the amplification of mitochondrial gene ATP6 (Table 1) with the following custom parameters:

- *Primers* melting temperature (Tm) of 62 °C, GC content of 50%, and 22 bp length.
- *Probe* Tm of 68 °C, GC content of 50%, and 24 bp length.

**Table 1:** Primer pairs and probes used in the study, the coding region, the primer sequence, the melting temperature, and the amplicon size.

Primer name	Assay	Primers	Region	Sequence 5ʹ–3ʹ	Tm (°C)	Product (bp)
Tpit_cF	cPCR	_c_F	TRNK-ATP8	TCTAATGAAACTATTAACAC	48	131
Tpit_cR		_c_R		ATAATAATCAATTAATGGGC	48	
Tpit_qF	qPCR	_q_F	ATP6	ATTATTCGACCCGGTACTTTGG	62	89
Tpit_qR		_q_R		ATAACTCTCTTAAGAAGAACAGGACC	62	
Tpit_qP		_q_P		ACGATTAACAGCAAACATAATTGCCGGAC	68	

The specificity of the assemblage of primers and probes suggested by the software was checked and verified in silico against available sequence data (https://www.ncbi.nlm.nih.gov/genbank/). We selected the primers, probe and amplicon sequences specific to *T. pityocampa* (100% similarity value, Table 1).

### DNA sampling

Primers were empirically tested in both fresh *T. pityocampa* samples and bat faecal samples to test their efficiency by both PCR techniques.

Fresh *T. pityocampa* male moths were collected from the Basque Country (Southwestern Europe) using pheromone-baited G-traps (Econex, Murcia, Spain). Traps were suspended from trees at the height of ~ 4 m. Additionally, we placed a couple of light traps during a single night to capture moths other than *T. pityocampa*. Insects were then collected and stored at − 80 °C until processed. A couple of hind legs from individuals of *T. pityocampa* and other moth species were used for DNA extraction (Table S1). The whole extraction process was carried out according to the protocol described by Ref.^41^ with slight modifications. In fact, after washing the DNA pellets with 70% ethanol, we centrifuged them for 10 min, 4 °C at 13,000 rpm, dried them at 60 °C for 30 min and resuspended them in 30 μL ddH_2_0 overnight. Likewise, we collected bat droppings in 2014, 2016, 2017 and 2018 underneath bat colony clusters of different species (*Myotis crypticus*, *M. daubentonii*, *M. emarginatus*, *Miniopterus schreibersii*, *Pipistrellus kuhlii*, *P. pipistrellus*, *Rhinolophus euryale*, *R. hipposideros*, *R. ferrumequinum*-*M. emarginatus* and *Tadarida teniotis*) throughout different Iberian Peninsula regions (Basque Country and Andalusia, Fig. S1). Stools were then dried at 40 °C and stored at − 80 °C until processed. No animal ethics clearance was required for this study because samples were passively and noninvasively collected underneath bat colony clusters, not involving manipulation of endangered or legally protected species.

DNA from these faecal samples was extracted as explained in Ref.^17^, and extraction products were stored at − 20 °C. We included an extraction control with each round. An aliquot from these DNA extracts was further analysed through metabarcoding according to the protocol described by Ref.^15^. The aforementioned samples were classified as follows (for further information, see Table S1):

1. *C*+, DNA samples from fresh *T. pityocampa* (positive controls),
2. *C−*, DNA samples from fresh moths other than *T. pityocampa* (negative controls),
3. *Th*+, DNA samples from bat faeces containing traces of *T. pityocampa* according to metabarcoding (sequences matching 98.5% similarity value^42^ exclusively with *T. pityocampa* were considered),
4. *Th−*, DNA samples from bat faeces in which traces of the pest were not found.

Similarly, we aimed to evaluate whether the developed PCR assays are more sensitive than metabarcoding at detecting *T. pityocampa.* Thus, we divided *Th− *samples into two subgroups:

4.1 *Th*_*out*_−, samples collected in non-forest habitats and out of the *T. pityocampa* flight period,
4.2 *Th*_*in*_−, samples collected within forest habitats throughout the *T. pityocampa* flight period.

### cPCR and qPCR

In the qPCR framework, A260/280 ratio is routinely used for purity assessment of DNA, which is an indication of its presence and quality^43^. Impure samples have ratios outside an acceptable range and they are therefore discarded from the analysis. Thus, in all, we selected 184 samples for analysis (*C*+, n = 6; *C−*, n = 18; *Th*+ = 96; *Th−* = 63), but only the samples meeting some purity criteria were retained (A260/A280 ratio 1.77 ± 0.24; n = 169). We only kept quantification data from qPCR runs with R^2^ values of ≥ 0.98 and slopes between 3.19 and 3.71, which indicated 86–105% efficiency of qPCR reactions.

#### Qualitative assessment

To validate the detection efficiency of both PCR techniques, the size of each sample group was as follows (Table S1):

- *C*+: 5 samples;
- *C−*: 7 samples;
- *Th*+: 83 samples;
- *Th*_*out*_−: 22 samples);
- *Th*_*in*_−: 21 samples).

We assayed each sample in triplicate, and we included a negative PCR with each round. For cPCR, we compared several PCR protocols and master mixes in an initial pilot study, and finally, we selected the KAPA HiFi HotStart ReadyMix PCR Kit, which performed best. The amplifications were conducted in 25 μL containing 12.5 μL KAPA HiFi ReadyMix, 1.25 μL of each primer (10 μM), 9 μL Milli-Q H_2_O and 1 μL of template DNA per sample. The reaction conditions included: 95 °C for 3 min, followed by 10 cycles of 95 °C for 30 s, 50 °C (− 0.5 °C/cycle ramp) for 30 s and 72 °C for 30 s, 25 cycles of 95 °C for 30 s, 48 °C for 30 s, 72 °C for 30 s and ended by 72 °C for 10 min. PCR products were migrated with a DNA 5 K Reagent kit in a LabChip GX Touch 24 Nucleic Acid Analyzer. For qPCR, before amplification processing, we quantified DNA from each sample by a NanoDrop 8000 and based on this quantification, we adjusted the DNA concentration to 10 ng/μL. Afterward, qPCR reactions were performed in a CFX96 Touch Real-Time PCR system (Bio-Rad) using 5 μL NZY qPCR Probe Master Mix (2x) mixture, 0.4 μL of each primer (10 mM), 0.1 μL of probe (10 mM), 3.1 μL nuclease-free H_2_O and 1 μL template DNA for a total volume of 10 μL. The amplification conditions were: 95 °C for 10 min, followed by 40 cycles of 95 °C for 15 s and 62 °C for 45 s. In order to test primers’ specificity, some PCR products (cPCR, n = 5; qPCR, n = 7) were purified and sequenced in forward and reverse directions using either an ABI3730XL or an ABI3700 Genetic Analyser (Applied Biosystems). The resulting forward and reverse sequences were assembled using BioEdit 7.1^44^ or Geneious 8.0.2^45^ and checked for errors and ambiguities.

#### Quantitative assessment

**Figure 1:**
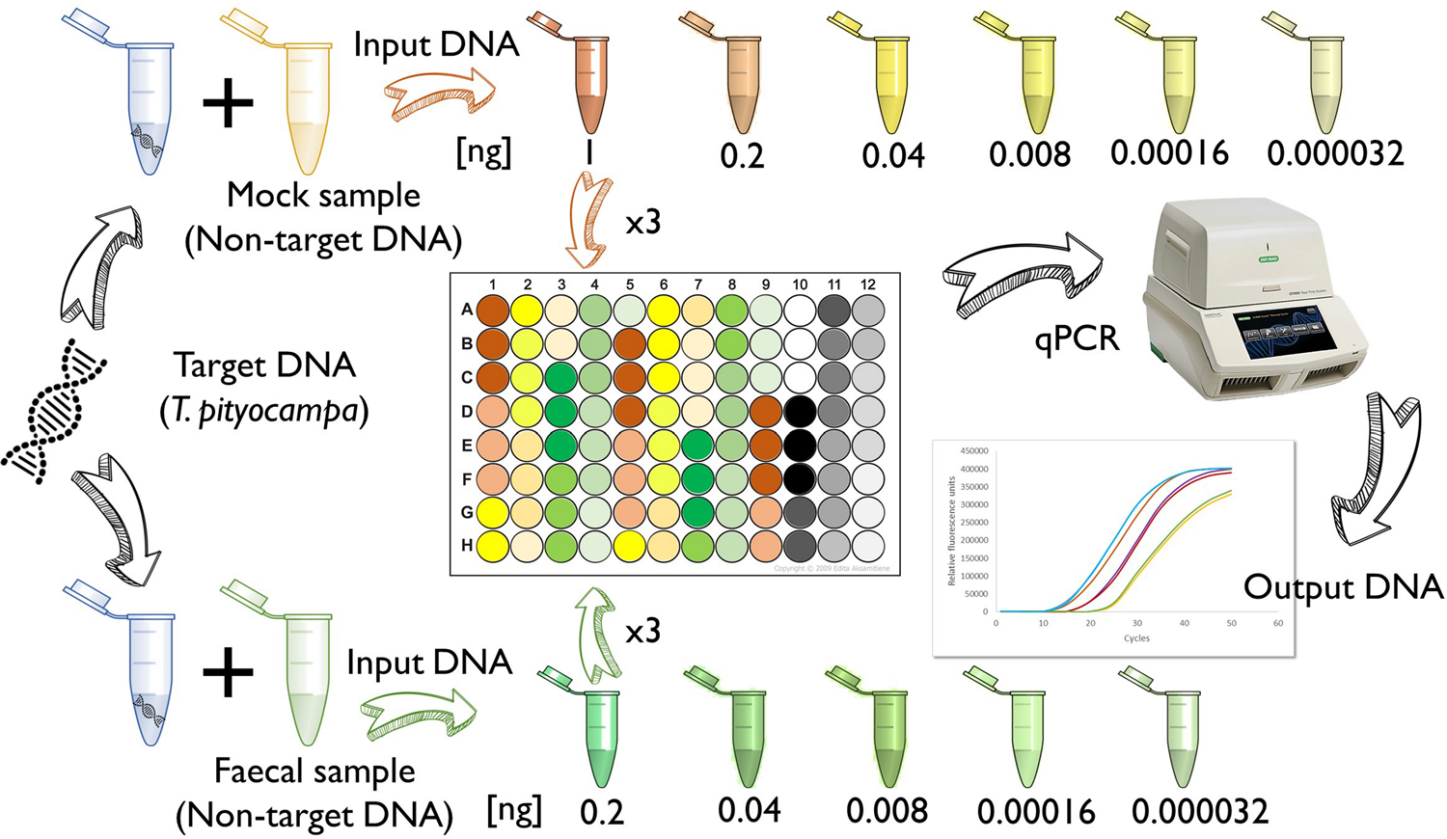
Overview of the experimental workflow for the quantitative assessment of qPCR.

We assessed qPCR’s quantification accuracy in two types of media: moths mocks and bat faecal samples (Table S2). We prepared mock mixtures composed of DNA from combinations of seven common prey species of bats (*Lobesia botrana* and *Cydia funebrana* [Tortricidae], *Synthymia fixa*, *Catocala nymphagoga*, *Lymantria dispar*, *Noctua pronuba*, *Eremobia ochroleuca* [Noctuidae]) with equal amounts of DNA. We also incorporated some of the faecal samples collected in non-forest habitats in which DNA traces of *T. pityocampa* were not found by metabarcoding (*Th*_*out*_−) belonging to different bat species (i.e., *Rhinolophus hipposideros*, *Pipistrellus pipistrellus*, *P. kuhlii*, *Myotis daubentonii*, Fig. S1), to include DNA of a broader range of prey associated with the particular feeding habits of each bat species. DNA of samples was quantified by a NanoDrop 8000, and subsequently, it was diluted to adjust DNA concentrations to specific gradients (assays with 1, 0.2, 0.04, 0.008, 0.00016 and 0.000032 ng/µL DNA, Fig. 1). Finally, we empirically compared known concentrations of target DNA (hereafter called “input DNA”) and estimated concentrations by qPCR (hereafter called “output DNA”).

For the experiment, we tested (1) 29 mock sample mixtures, i.e., fresh DNA from *T. pityocampa* (*C*+) mixed with DNA of other moths (*C−*) and (2) 35 faecal sample mixtures, mixed DNA from fresh *T. pityocampa* (*C*+) and bat faeces without *T. pityocampa* (*Th*_*out*_−) (Fig. 1). In faecal mixtures, *T. pityocampa* DNA concentration of 1 ng/µL was not used due to problems in maintaining molarity values. The amplification process was carried out following the same qPCR protocol described above. Besides, we also constructed standard curves in triplicate using a five-fold dilution series of targeted templates, from 3.3 × 10^6^ to 5.2 × 10^10^ copies per μL, to predict the abundance of target DNA present in tested samples.

### Data analysis

All the statistical analyses were carried out in R Studio v. 1.4.1103 and R v.4.0.3.^46^. We conducted further analysis for each of the following questions:

#### The effect of replication effort on detection probabilities

As described by Ref.^27^, an increase in the PCR replication effort leads to an increased likelihood of detecting a target present in the sample. Accordingly, we aimed to evaluate the detection probabilities over the three replicates, for which we used the following equation for each of the replicates:

$${\text{D}}/\left( {{\text{D}} + {\text{ND}}} \right),$$

where D is the number of detections and ND is the number of non-detections.

#### The relationship between DNA quantity and detection probability by cPCR and qPCR

We should expect a higher detection rate in those samples with a higher target DNA concentration. Hence, we examined if there is a relationship between the average DNA abundance estimated from triplicates of *Th*+ samples by qPCR and the detection rates by each PCR assay over the three replicates, for which we fitted logistic regression models (quasi-binomial family) using *glm* function of the *stats* package in R^46^.

#### The relationship between input and output DNA concentrations

We tested whether the input DNA concentrations correlated with the average concentrations estimated by triplicated samples in both mock and faecal samples and determined our qPCR technique’s accuracy to predict target DNA concentrations. For that, we compared abundances yielded by qPCR against the expected DNA concentrations. Ideally, we should expect a positive correlation with a slope near 1. Hence, we initially fitted a linear regression model, but the residual spread increased along with the explanatory variable, which violated the homogeneity of variance assumption of our linear regressions. Therefore, we fitted a generalised least squares (GLS) model with a combined variance structure using the *varComb* function of the *nlme* package^47^, allowing an increase in the residual spread for larger input DNA concentrations as well as a different spread per sample type (mock or faecal). Afterwards, we carried out a likelihood ratio test for the fixed component selection, comparing nested models with three possible explanatory variables: run, sample type and input DNA. The test indicated that neither run nor sample type were important variables affecting the yielded DNA by qPCR. Therefore, the final model only included input DNA as an explanatory variable. We plotted all the models using the *ggplot2* package^48^.

## Results

Sequence analysis of the qPCR products revealed that all the *T. pityocampa* amplicons were 100% identical to the expected 89 bp fragment (Fig. S2). cPCR products also exclusively matched to *T. pityocampa,* but they did not show the same consistency as qPCR. Indeed, only two reverse sequences from cPCR sequencing analysis were 99% and 95% similar to the 131 bp fragment sequence, respectively. The rest of the sequences were not long enough to assess the adscription of the fragment.

**Figure 2:**
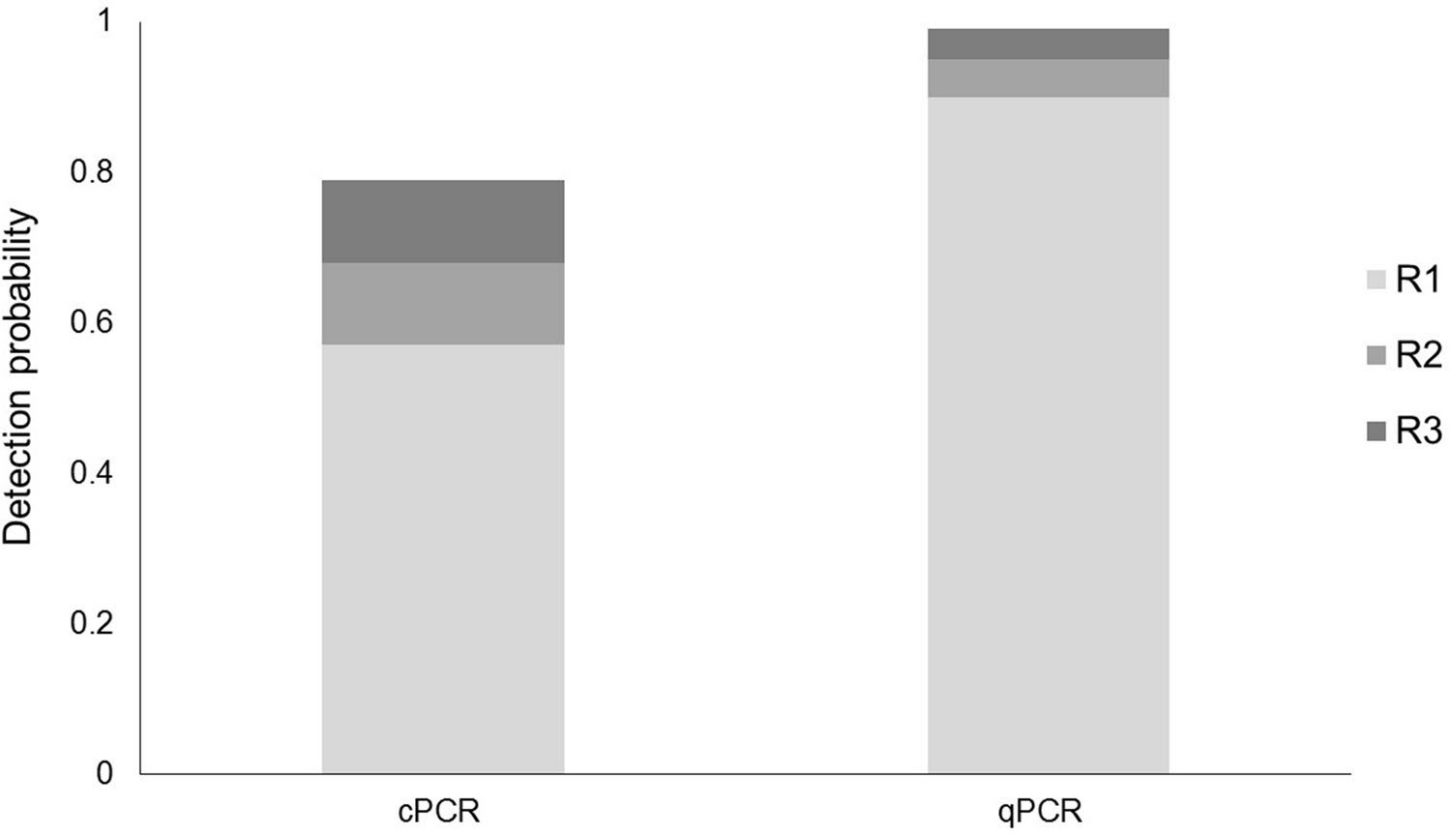
Detection probability of *T. pityocampa* DNA traces in faecal samples for each PCR assay as we increased the replicate effort (R).

Overall, none of the negative controls showed signs of amplification and all the positive controls and their corresponding replicates exhibited amplification signals in both PCR assays (Fig. S3, Table S1). cPCR showed a lower detection rate than qPCR for *Th* + samples (Fig. 2), besides the increasing number of test replicates had a bigger impact on the detectability rate of the cPCR than the qPCR. Both assays tested negative for *Th*_*out*_* − *samples, but some *Th*_*in*_*− *samples resulted in positive DNA signals (Table S1). Further, some samples tested with cPCR showed some nonspecific bands of different DNA fragment lengths together with the targeted band (Table S1).

**Figure 3:**
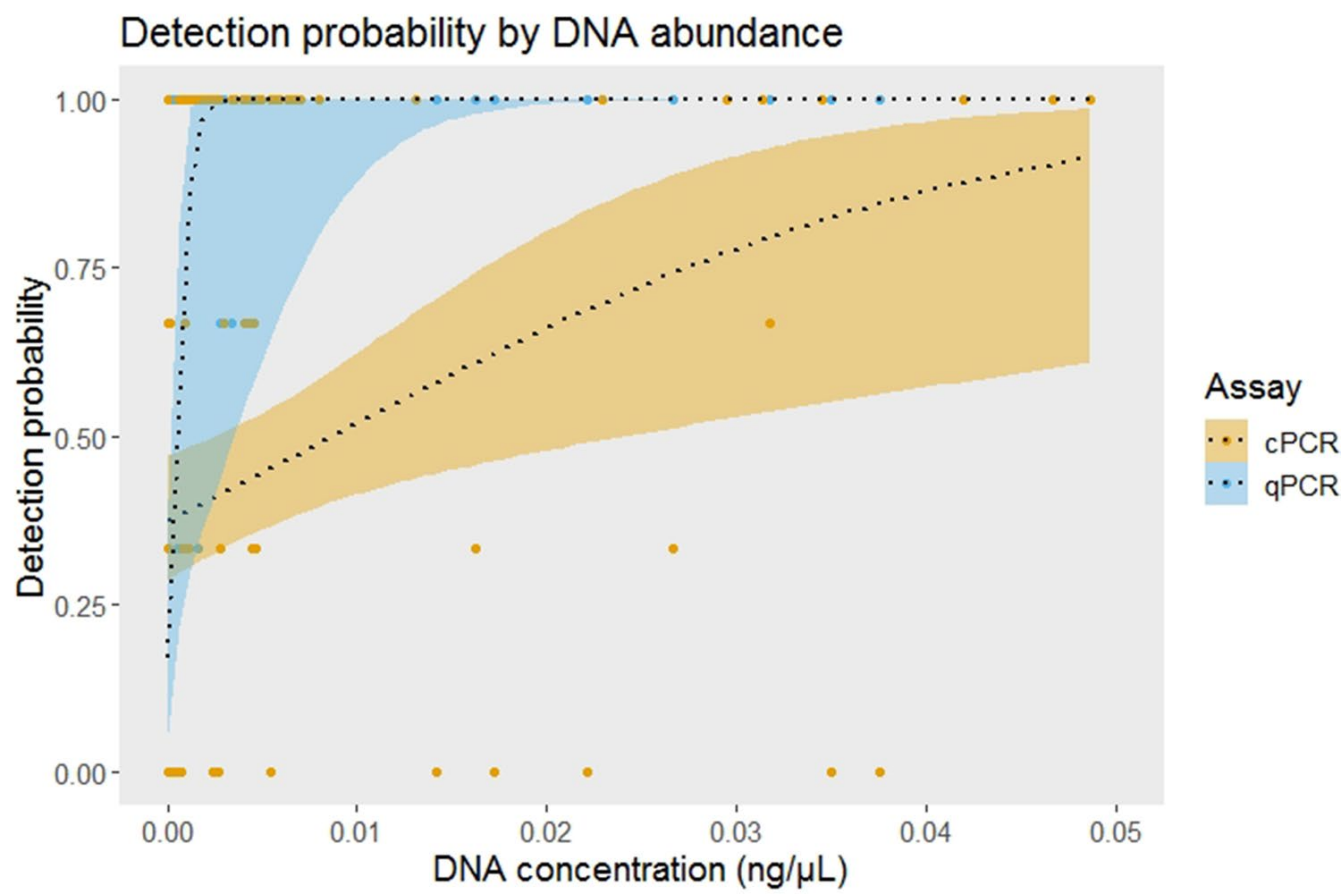
The relationship between the detection probability and DNA concentration for cPCR and qPCR. The dotted lines indicate the fitted regression model and the coloured bands the 95% confidence interval.

Higher DNA concentrations of *T. pityocampa* in *Th*+ samples resulted in an increased detection probability by the qPCR (t = 2.21, p < 0.05), whereas that correlation did not hold for the cPCR assay (t = 1.16, p = 0.25) (Fig. 3).

**Figure 4:**
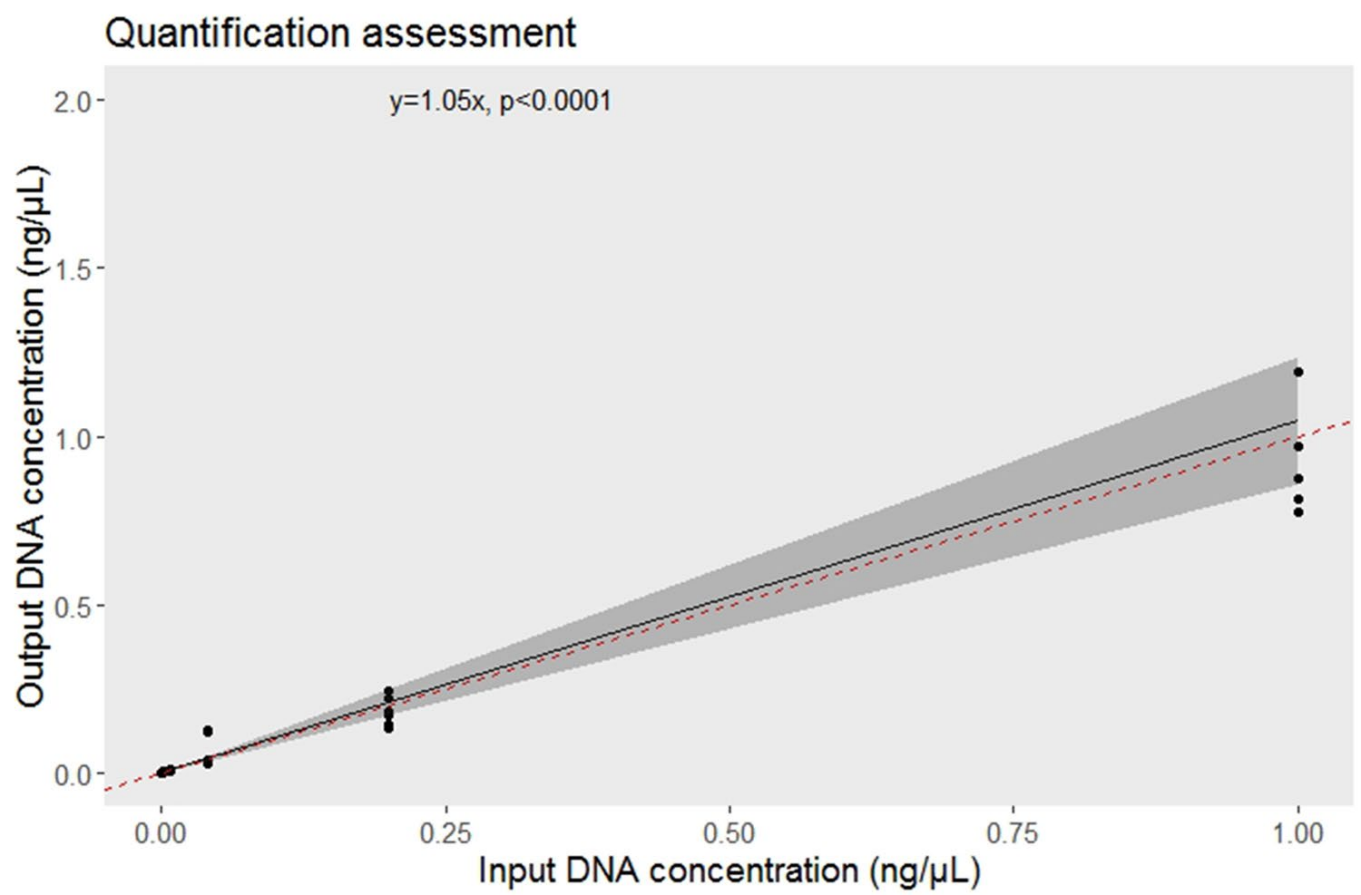
The relationship between the input and output DNA concentration. The grey band indicates the 95% confidence interval and the dotted red line represents a slope of 1.

The results from the quantitative assessment experiment showed there is a highly significant correlation between the input and output DNA concentrations (t = 10.84, p < 0.001) with a mean slope value of 1.05 (95% CI 0.85 to 1.23) (Fig. 4). This output indicated that the average DNA concentration estimated from qPCR triplicates represented the DNA concentration present in the sample within a range of 85% and 123%.

## Discussion

We observed that the performance of two primer sets and PCR methods differed, affecting their efficiency to detect DNA traces of *T. pityocampa* in bat faeces. Studies on predator–prey trophic interactions are currently among the main highlights of ecological research (e.g.^42,49–51^). In this context, finding robust molecular methods to reliably identify and quantify DNA of prey remains in predators’ faeces is a fundamental issue, for instance, to gain insight into the ecosystem services provided by predators as pest suppressors.

Both PCR assays exclusively identified *T. pityocampa,* as revealed by the sequencing analysis, indicating they are highly specific to our target. In some samples, though, cPCR produced a predominant target band of 131 bp with multiple nonspecific bands of different DNA fragment length, which suggests a reduction in the efficiency of the amplification, likely due to non-optimal amplification conditions, such as sub-optimal annealing temperatures^52^, MgCl2 concentration, primer concentration, or PCR cycling conditions^53^.

### Comparison of cPCR and qPCR

In line with previous findings of^27,54,55^, qPCR had a higher sensitivity than cPCR. It proved to be a more reliable and robust assay to detect *T. pityocampa* in bat faecal samples for two main reasons.

#### Replication effort

Increasing the number of replicates leads to an improvement in the DNA detection probability. Nonetheless, while cPCR requires up to three replicates to detect *T. pityocampa* in the 80% of *Th*+ samples, qPCR detected the target in 90% of the samples with a single replicate and 99% of them with three replicates. The only sample not tested positive by any of the PCR assays was processed in 2014, and only 8 copies of the target sequence were found that time by metabarcoding. Hence, we cannot discard that the DNA degradation in such an old sample might have hampered the detection of *T. pityocampa*.

#### Detectability and DNA concentration

Ideally, a robust PCR method for species monitoring should reliably detect the target species, increasing the detection probability as the target DNA concentration in the sample rises^56^. In line with this, the qPCR assay results showed a positive relationship between DNA concentration and the detection probability, which suggests that a higher incidence of *T. pityocampa* in bats’ diet entails a greater likelihood of detection. In contrast, the lack of such correlation in cPCR denotes that the detectability of *T. pityocampa* might be influenced by other disturbing factors, such as the presence of PCR inhibitors in some samples. In fact, they affect more severely to cPCR than qPCR owing to its less sensitive detection mechanisms^54,57^, which in turn, prompt false-negative results^58^, and a higher unpredictability.

### Diet quantification by qPCR

Our study also demonstrates the potential of qPCR assays to quantify DNA in bat faecal samples and, as a result, infer the predation levels on specific prey. We undertook extensive validation steps using mocks and faecal samples from a vast array of bat species, suggesting that the developed qPCR assay is robust for quantifying *T. pityocampa* in the bat faeces irrespective of the sample source. Further methodological studies should focus on developing or improving current molecular tools for the simultaneous identification and quantification of highly diverse polyphagous predators’ diets, where dozens of species occur at a time (e.g.^17^). The framework of this study can be taken as a model to design similar assays applicable to other species of interest, such as agricultural pests or insects of public health concern. Further, the incorporation of the multiplex qPCR, including Taq-man probes with various dyes, will enable the simultaneous amplification of multiple prey targets in a single reaction (Ref.^59^; e.g., up to five species with the CFX96 Real-Time PCR Detection System used in the study). Because of the affinity differences between primers and their target sequences^28^, quantitative interpretations from dietary data must focus on the intraspecific variations between samples rather than on interspecific differences.

### Decision making

Currently, the two most common approaches to detect DNA from environmental samples rely typically on using metabarcoding with Next Generation Sequencing (NGS) for the simultaneous identification of multiple taxa or on using targeted PCR approaches through species-specific primers and probes. Deciding on which one to use depends on the research question and available economic and personnel resources. The advantages, limitations and usage of each technique are further discussed (Table 2).

**Table 2:** Advantages, limitations, usage and costs of the molecular techniques used in the study.

Technique	Advantages	Limitations	Usage	Costs
Metabarcoding	Wide dietary breadth. No need for a priori knowledge of the diet Multiple samples per run (≈384) Universal primers that are usually predesigned	Less sensitive than qPCR for single species detection Target organisms must be in the reference databases Expensive equipment and reagents High workload (library preparation, sequencing, bioinformatics)	Diverse diet studies Complex trophic networks	5.30–8.60€/replicate
cPCR	Specific Low-cost (equipment and reagents)	Post-PCR (time consuming, possible cross-contamination) Low sensitivity Three replicates, at least Qualitative assay (presence-absence) Specific primers for a target pest may not always be possible	Pilot studies: primary step to evaluate the interest of any further analysis Overall screening at a low price	0.17€/replicate
qPCR	Highly specific and sensitive Rapid analysis (No post-PCR) Qualitative and quantitative assay Multiple samples per run (≈120 in a 384-well plate with three replicates)	High costs (equipment and reagents) Specific primer and probes for a target pest may not always be possible	Relative levels of predation on particular prey species	1.50€/replicate

### Metabarcoding

Metabarcoding allows the parallel processing and sequencing of several hundred samples per run^60^. Thus, in the last decade, metabarcoding has emerged as an invaluable molecular tool for characterising diet breadth in polyphagous predators. It offers unique opportunities for deciphering trophic interactions within food webs^61^. Nonetheless, this gain in trophic network understanding comes at the cost of accuracy and sensitivity for target species detection^62^. For instance, the results of our study revealed that more than 10% of the samples which returned no metabarcoding reads that were assigned to *T. pityocampa*, contain the species when using cPCR or qPCR (Table S1). Therefore, if a study requires information on a species-rich or unknown diet, metabarcoding is likely the appropriate technique. However, targeted approaches can be superior if there are specific prey items of interest, quantification of these prey is desired, or rapid and cost-effective analyses are required.

### cPCR

The results from the cPCR revealed that the assay is 80% effective as long as three replicates per sample are used. Further, augmenting the replication effort may lead to an increase in the detection rate of *T. pityocampa*, as shown in Fig. 2. The technique can be beneficial for pilot studies, for instance, as a primary step to evaluate the interest of any further analysis, or even for timely detection of *T. pityocampa* in bat faeces before pest population settlement^63^, which offer valuable information for pest management to avoid further irreparable damage to the pine stands. In addition, one of the main advantages of this assay lies in its low price (e.g., 0.17€/replicate^64^) compared with qPCR (1.50€/replicate^64^) or metabarcoding (5.30–8.60€/replicate^65^), which makes it affordable for almost any research study. However, we do not recommend the assay for exhaustive monitoring in studies requiring high sensitivity.

### qPCR

The qPCR assay offers a fined-grained qualitative and quantitative assessment of *T. pityocampa* in bats’ diet. As such, the approach has a dual-use as it can be used for both presence-absence monitoring and as an approximation of *T. pityocampa* biomass consumed by bats. In addition, a single replicate produces still a 90% detection rate and consequently, costs per sample may be reduced. For quantification purposes though, there is considerable variability between replicates and we must keep at least three replicates per sample to ensure the reliability of the quantitative assessment.

## Supplementary Information

Supplementary Information.

## Data Availability

All the data are available in the manuscript or Supplementary Material.
