# Supplementary material for: Molecular assays to reliably detect and quantify predation on a forest pest in bats faeces
Source: Sci Rep. 2022 Feb 10;12:2243. doi: 10.1038/s41598-022-06195-7 (PMC8831491; doi:10.1038/s41598-022-06195-7)
Supplement: Supplementary file 1 — Supplementary Information. [file 41598_2022_6195_MOESM1_ESM.pdf]

Supplementary Material

Molecular assays to reliably detect and quantify predation on a forest pest in bats faeces

Unai Baroja, Inazio Garin, Nerea Vallejo, Amaia Caro, Carlos Ibáñez, Andrea Basso, Urtzi Goiti.

Corresponding author: Unai Baroja; **E-mail:** unai.baroja@ehu.eus

This supplementary material contains:

- **Supplementary information.** Protocol used for moth DNA extraction.
- **Table S1**. Samples used in the study, their corresponding group, the species name and the presence (+)/absence (-) outcome with Metabarcoding and PCR methods through different replicates (R).
- **Table S2.** Samples used for the quantitative assessment, the type of sample and input and output concentration of *T. pityocampa* through different replicates (R).
- **Figure S1.** Geographic distribution of sampling locations and the number of samples used for qualitative assessment.
- **Figure S2.** DNA sequencing results for the qPCR products: all amplicons corresponded to the same *T. pityocampa* sequence.
- **Figure S3.** Amplification bands of cPCR products revealed by the LabChip GX Touch nucleic acid analyzer.

Supplementary information. Protocol used for moth DNA extraction.

We perform the following steps in a **laminar flow cabinet.**

1.- <u>Digestion</u>

1.1. Place samples (2 moth legs/…) into a 2 ml sterile tube.
1.2. Add **400 µl Digestion Buffer** to the tube.
1.3. Add silica balls.
1.4. **Homogenize** using a Polytron Tissue Homogenizer, for 20 seg (this process could be **repeated twice**).
1.5. Add **40 µl of 20% SDS** (2% final concentration).
1.6. Add **8 µl of 20 mg/ml protease** K (400 µg/ml final concentration). Mix well.
1.7. **Incubate at 55-65º C** overnight (RPM=85).

2.- <u>DNA extraction</u>

2.1. Add **300 µl 6 M NaCl** to each mixture sample.

2.2. Mix (**vortex**) samples for **30 seg** at maximum speed.

2.3. Spun down (**centrifugate**) tubes for **30 min** at 10000 G-force = rcf (9703 rpm).

2.4. Transfer the **supernatant** (the upper fase) to 1.5 ml fresh tubes (avoid interphase).

3.- <u>DNA precipitation</u>

3.1. Add an equal volume of **isopropanol** to each sample. Mix well (by hand).

3.2. Incubate: -20º C for 1 h

3.3. Centrifugate (20 min, 4º C, 10000 G-force = rcf (9703 rpm)).

3.4. Remove alcohol with a pipette → better to leave some alcohol in the tube rather than to suck off the pellet.

4.- <u>DNA washing</u>

4.1. Add 700 µl 70% Ethanol to the tube.

4.2. Centrifugate (10 min, 13000 rpm, 4 ºC).

4.3. Remove alcohol with a pipette.

4.4. Evaporate with the tube opened → into a closed and previously washed “rack” to avoid DNA contamination. - 60º C for 30 min

4.5. Add 30 µl ddH_2O to the DNA pellet.

4.6. Incubate at room temperature at least for 5 h (better overnight) to resuspend pellet.

Table S1. Samples used in the study, their corresponding group, the species name and the presence (+)/absence (-) outcome with Metabarcoding and PCR methods through different replicates (R).

					cPCR			qPCR		
**Sample**	**Group**		**Species**	**Metabarcoding**	R^1	R^2	R^3	R^1	R^2	R^3
Th1	C+		Tpi	NA	+	+	+	+	+	+
Th2	C+		Tpi	NA	+	+	+	+	+	+
Th3	C+		Tpi	NA	+	+	+	+	+	+
Th4	C+		Tpi	NA	+	+	+	+	+	+
Th5	C+		Tpi	NA	NA	NA	NA	+	+	+
Lep1	C-		*Sfix*	NA	-	-	-	-	-	-
Lep2	C-		*Cnym*	NA	-	-	-	-	-	-
Lep3	C-		*Cnym*	NA	-	-	-	-	-	-
Lep4	C-		*Ldisp*	NA	-	-	-	-	-	-
Lep5	C-		*Ldisp*	NA	-	-	-	-	-	-
Lep6	C-		*Npro*	NA	-	-	-	-	-	-
Lep7	C-		*Eocr*	NA	-	-	-	-	-	-
619va9	Th^+		Msc	+	-	-	-	+	+	+
621msc10	Th^+		Msc	+	-	-	-	+	+	+
621msc4	Th^+		Msc	+	+	+	+	+	+	+
621msc5	Th^+		Msc	+	-	+	+	+	+	+
621msc8	Th^+		Msc	+	+	+	+	+	+	+
717va1	Th^+		Msc	+	+	+	+	+	+	+
717va10	Th^+		Msc	+	-	-	-	+	+	+
717va2	Th^+		Msc	+	+	+	+	+	+	+
717va3	Th^+		Msc	+	-	-	-	+	+	+
717va4	Th^+		Msc	+	-	-	-	+	+	+
717va6	Th^+		Msc	+	-	-	-	+	+	+
717va7	Th^+		Msc	+	-	-	-	+	+	+
717va8	Th^+		Msc	+	-	-	-	+	+	+
717va9	Th^+		Msc	+	-	+	+	+	+	+
720mor11	Th^+		Msc	+	+	+	+	+	+	+
720mor2	Th^+		Msc	+	+	+	+	+	+	+
720mor3	Th^+		Msc	+	+	+	+	+	+	+
720mor4	Th^+		Msc	+	-	+	+	+	+	+
720mor5	Th^+		Msc	+	-	+	+	+	+	+
720mor7	Th^+		Msc	+	+	+	+	+	+	+
720mor9	Th^+		Msc	+	+	+	+	+	+	+
728mor1	Th^+		Msc	+	-	-	+	+	+	+
728mor10	Th^+		Msc	+	+	+	+	+	+	+
728mor3	Th^+		Msc	+	+	+	+	+	+	+
728mor4	Th^+		Msc	+	+	+	+	+	+	+
728mor5	Th^+		Msc	+	-	+	+	+	+	+
728mor8	Th^+		Msc	+	+	+	+	+	+	+
728mor9	Th^+		Msc	+	+	+	+	+	+	+
730msc1	Th^+		Msc	+	-	+	+	+	+	+
731va1	Th^+		Msc	+	+	+	+	+	+	+
731va10	Th^+		Msc	+	-	-	+	-	+	+
731va2	Th^+		Msc	+	+	+	+	+	+	+
731va5	Th^+		Msc	+	-	-	+	+	+	+
731va6	Th^+		Msc	+	-	-	+	+	+	+
73mor10	Th^+		Msc	+	+	+	+	+	+	+
73mor2	Th^+		Msc	+	+	+	+	+	+	+

Table S1. (continuation).

				cPCR			qPCR		
**Sample**	**Group**	**Species**	**Metabarcoding**	**R^1**	**R^2**	**R^3**	**R^1**	**R^2**	**R^3**
73mor3	Th$^+$	Msc	+	+	+	+	+	+	+
73mor5	Th$^+$	Msc	+	+	+	+	+	+	+
73mor7	Th$^+$	Msc	+	+	+	+	+	+	+
73mor9	Th$^+$	Msc	+	+	+	+	+	+	+
76msc2	Th$^+$	Msc	+	-	-	+	+	+	+
76msc6	Th$^+$	Msc	+	+	+	+	+	+	+
816 mor9	Th$^+$	Msc	+	+	+	+	-	-	+
816mor1	Th$^+$	Msc	+	+	+	+	+	+	+
816mor2	Th$^+$	Msc	+	-	-	-	+	+	+
816mor3	Th$^+$	Msc	+	-	+	+	+	+	+
816mor4	Th$^+$	Msc	+	+	+	+	+	+	+
816mor5	Th$^+$	Msc	+	+	+	+	+	+	+
816mor6	Th$^+$	Msc	+	+	+	+	+	+	+
816msc13	Th$^+$	Msc	+	+	+	NA	+	+	+
817va10	Th$^+$	Msc	+	+	+	+	+	+	+
817va5	Th$^+$	Msc	+	+	+	+	+	+	+
817va6	Th$^+$	Msc	+	-	-	+	+	+	+
829 mor1	Th$^+$	Msc	+	+	+	+	-	+	+
829 mor3	Th$^+$	Msc	+	+	+	+	+	+	+
829 mor5	Th$^+$	Msc	+	+	+	+	+	+	+
829 mor6	Th$^+$	Msc	+	+	+	+	+	+	+
829 mor7	Th$^+$	Msc	+	+	+	+	+	+	+
829 mor8	Th$^+$	Msc	+	+	+	+	+	+	+
829 mor9	Th$^+$	Msc	+	+	+	+	+	+	+
911 mor2	Th$^+$	Msc	+	+	+	+	+	+	NA
915 va9	Th$^+$	Msc	+	+	+	+	+	+	+
721reu2	Th$^+$	Reu	+	-	-	-	-	-	-
721reu3	Th$^+$	Reu	+	+	+	+	+	+	+
721reu4	Th$^+$	Reu	+	NA	NA	NA	+	+	+
721reu8	Th$^+$	Reu	+	+	+	+	+	+	+
77reu2	Th$^+$	Reu	+	+	+	+	+	+	+
77reu3	Th$^+$	Reu	+	+	+	+	+	+	+
77reu5	Th$^+$	Reu	+	+	+	+	+	+	+
77reu6	Th$^+$	Reu	+	+	+	+	+	+	+
721ajd	Th$^+$	Rfe&Mem	+	-	-	+	-	+	+
817spa	Th$^+$	Rfe&Mem	+	-	+	+	+	+	+
824an	Th$^+$	Rfe&Mem	+	-	-	-	+	+	+
831bad	Th$^+$	Rfe&Mem	+	-	-	-	+	+	+
831spc	Th$^+$	Rfe&Mem	+	-	-	-	+	+	+
83baa	Th$^+$	Rfe&Mem	+	-	-	-	+	+	+
83bab	Th$^+$	Rfe&Mem	+	-	-	-	+	+	+
96an	Th$^+$	Rfe&Mem	+	-	-	-	+	+	+
717tte1	Th$^+$	Tte	+	-	+	+	+	+	+
717tte2	Th$^+$	Tte	+	-	-	+	-	-	+
717tte6	Th$^+$	Tte	+	+	+	+	+	+	+
717tte8	Th$^+$	Tte	+	-	-	+	+	+	+
717tte9	Th$^+$	Tte	+	-	-	-	+	+	+
621mab	Th^{-OUT}	Mda	-	-	-	-	-	-	-
621mac	Th^{-OUT}	Mda	-	-	-	-	-	-	-
621mv	Th^{-OUT}	Pku	-	-	-	-	-	-	-
531ab	Th^{-OUT}	Ppi	-	-	-	-	-	-	-

Table S1. (continuation).

Sample	Group	Species	Metabarcoding	cPCR R^1	cPCR R^2	cPCR R^3	qPCR R^1	qPCR R^2	qPCR R^3
621ab	Th^{-OUT}	Ppi	-	-	-	-	-	-	-
67ab	Th^{-OUT}	Ppi	-	-	-	-	-	-	-
510sa	Th^{-OUT}	Ppi	-	-	-	-	-	-	-
523sa	Th^{-OUT}	Ppi	-	-	-	-	-	-	-
621sa	Th^{-OUT}	Ppi	-	-	-	-	-	-	-
68sa	Th^{-OUT}	Ppi	-	-	-	-	-	-	-
510vel	Th^{-OUT}	Ppi	-	-	-	-	-	-	-
531vel	Th^{-OUT}	Ppi	-	-	-	-	-	-	-
621vel	Th^{-OUT}	Ppi	-	-	-	-	-	-	-
68vel	Th^{-OUT}	Ppi	-	-	-	-	-	-	-
620haa	Th^{-OUT}	Rhi	-	-	-	-	-	-	-
621le	Th^{-OUT}	Rhi	-	-	-	-	-	-	-
531rva	Th^{-OUT}	Rhi	-	-	-	-	-	-	-
531rvb	Th^{-OUT}	Rhi	-	-	-	-	-	-	-
620rva	Th^{-OUT}	Rhi	-	-	-	-	-	-	-
620rvb	Th^{-OUT}	Rhi	-	-	-	-	-	-	-
67rva	Th^{-OUT}	Rhi	-	-	-	-	-	-	-
67rvb	Th^{-OUT}	Rhi	-	-	-	-	-	-	-
817fra	Th^{-IN}	Mcry	-	-	-	-	-	-	+
817lac	Th^{-IN}	Mem	-	-	-	-	-	-	-
522msc1	Th^{-IN}	Msc	-	-	-	-	-	-	-
522msc2	Th^{-IN}	Msc	-	-	-	-	-	-	-
522msc3	Th^{-IN}	Msc	-	-	-	-	-	-	-
69va2	Th^{-IN}	Msc	-	-	-	-	-	-	-
69va3	Th^{-IN}	Msc	-	-	-	-	-	-	-
924mor2	Th^{-IN}	Msc	-	+	+	+	+	+	+
924mor3	Th^{-IN}	Msc	-	-	+	+	-	-	-
924mor4	Th^{-IN}	Msc	-	-	+	+	+	+	+
924msc2	Th^{-IN}	Msc	-	-	-	-	-	-	-
924msc3	Th^{-IN}	Msc	-	-	-	-	-	-	-
713zea	Th^{-IN}	Pku	-	-	-	-	-	-	-
914ajc	Th^{-IN}	Rfe&Mem	-	-	-	-	-	-	+
615an	Th^{-IN}	Rfe&Mem	-	NA	NA	NA	-	-	-
622spa	Th^{-IN}	Rfe&Mem	-	-	-	-	-	-	-
622spd	Th^{-IN}	Rfe&Mem	-	-	-	-	-	-	-
720le	Th^{-IN}	Rfe&Reu	-	-	-	-	-	-	-
817ern	Th^{-IN}	Rhi	-	-	-	-	-	-	-
629go	Th^{-IN}	Rhi	-	-	-	-	-	-	-
824go	Th^{-IN}	Rhi	-	-	-	-	-	-	-

Group: C$^+$: TP samples; C$^-$: non-TP samples, Th$^+$: faecal sample in which TP was detected by NGS; Th^{-OFP}: faecal samples in which TP was not detected by NGS (out of TP flight period); Th^{-FP}: faecal samples in which TP was not detected by NGS (within TP flight period). **Species**: Tpi: *Thaumetopoea pityocampa*; Sfix: *Synthymia fixa*; Cnym: *Catocala nymphagoga;* Ldisp: *Lymantria dispar*; Npro: *Noctua pronuba*; Eocr: *Eremobia ochroleuca;* Mcry: *Myotis crypticus*; Mda: *M daubentonii*; Mem: *M. emarginatus*; Msc: *Miniopterus schreibersii*; Pku: *Pipistrellus kuhlii*; Ppi: *P. pipistrellus*; Reu: *Rhinolophus euryale*; Rfe: *R. ferrumequinum*; Rhi*: R. hipposideros*; Tte: *Tadarida teniotis.* NA: not available data.

Table S2. Samples used for the quantitative assessment, the type of sample, the species to which it belongs and input and output concentration of *T. pityocampa* through different replicates (R). Species, Tpi: *T. pityocampa*; Rhi: *R. hipposideros*; Pku: *P. kuhlii*; Ppi: *P. pipistrellus;* Mda: *M. daubentonii*; Various: the sample is composed by DNA of multiple species.

			[DNA]input (ng/µL)	[DNA]qPCR(ng/µL)			
**Sample**	**Type**	**Species**	**R^{123}**	**R^{1}**	**R^{2}**	**R^{3}**	**Mean**
Th6	Target	Tpi	5				
Th6	Target	Tpi	1				
Th6	Target	Tpi	0.2				
Th6	Target	Tpi	0.04				
Th6	Target	Tpi	0.008				
Th6	Target	Tpi	0.00016				
Th6	Target	Tpi	0.000032				
M1A^{1}	Mock	Various	1	0.61	0.63	1.67	**0.97**
M1A^{2}	Mock	Various	1	0.31	1.45	0.87	**0.88**
M1A^{3}	Mock	Various	1	0.92	0.22	1.30	**0.82**
M1B^{1}	Mock	Various	1	0.36	0.75	1.21	**0.77**
M1B^{2}	Mock	Various	1	1.38	0.18	2.01	**1.19**
M2A^{1}	Mock	Various	0.2	0.13	0.20	0.19	**0.17**
M2A^{2}	Mock	Various	0.2	0.17	0.04	0.34	**0.18**
M2A^{3}	Mock	Various	0.2	0.06	0.43	0.23	**0.24**
M2B^{1}	Mock	Various	0.2	0.03	0.10	0.28	**0.13**
M2B^{2}	Mock	Various	0.2	0.16	0.06	0.30	**0.17**
M3A^{1}	Mock	Various	0.04	0.018	0.036	0.068	**0.040**
M3A^{2}	Mock	Various	0.04	0.110	0.024	0.237	**0.124**
M3A^{3}	Mock	Various	0.04	0.029	0.223	0.130	**0.127**
M3B^{1}	Mock	Various	0.04	0.007	0.025	0.056	**0.029**
M3B^{2}	Mock	Various	0.04	0.023	0.006	0.069	**0.033**
M4A^{1}	Mock	Various	0.008	0.0040	0.0059	0.0135	**0.008**
M4A^{2}	Mock	Various	0.008	0.0056	0.0013	0.0156	**0.007**
M4A^{3}	Mock	Various	0.008	0.0024	0.0185	0.0072	**0.009**
M4B^{1}	Mock	Various	0.008	0.0018	0.0075	0.0088	**0.006**
M4B^{2}	Mock	Various	0.008	0.0106	0.0031	0.0194	**0.011**
M5A^{1}	Mock	Various	0.00016	0.00007	0.00007	0.00029	**0.00014**
M5A^{2}	Mock	Various	0.00016	0.00012	0.00001	0.00034	**0.00016**
M5A^{3}	Mock	Various	0.00016	0.00017	0.00007	0.00035	**0.00020**
M5B^{1}	Mock	Various	0.00016	0.00004	0.00008	0.00018	**0.00010**
M5B^{2}	Mock	Various	0.00016	0.00006	0.00024	0.00012	**0.00014**
M6A^{1}	Mock	Various	0.000032	0.000005	0.000032	0.000061	**0.000033**
M6A^{2}	Mock	Various	0.000032	-	0.000100	0.000060	**0.000082**
M6B^{1}	Mock	Various	0.000032	0.000041	0.000041	-	**0.000041**
M6B^{2}	Mock	Various	0.000032	-	0.000020	-	**0.000019**
074rvA^{1}	Faecal	Rhi	0.00016	0.00003	0.00002	0.00028	**0.00011**
074rvA^{2}	Faecal	Rhi	0.00016	0.00011	0.00035	0.00014	**0.00020**
074rvA^{3}	Faecal	Rhi	0.00016	0.00009	0.00032	0.00014	**0.00018**

074rvB^{1}	Faecal	Rhi	0.00016	0.00004	0.00013	0.00031	**0.00016**
074rvB^{2}	Faecal	Rhi	0.00016	0.00016	0.00044	0.00008	**0.00023**
074rvB^{3}	Faecal	Rhi	0.00016	0.00007	0.00033	0.00006	**0.00015**
074haA^{1}	Faecal	Rhi	0.00016	0.00007	0.00007	0.00026	**0.00013**
074haA^{2}	Faecal	Rhi	0.00016	0.00009	0.00052	0.00024	**0.00028**
074haA^{3}	Faecal	Rhi	0.00016	0.00007	0.00062	0.00020	**0.00030**
074haC^{1}	Faecal	Rhi	0.00016	0.00012	0.00013	0.00027	**0.00017**
074haC^{2}	Faecal	Rhi	0.00016	0.00006	0.00032	0.00019	**0.00019**

Table S2. (continuation).

			[DNA]input (ng/µL)	[DNA]qPCR(ng/µL)			
**Sample**	**Type**	**Species**	**R^{123}**	**R^{1}**	**R^{2}**	**R^{3}**	**Mean**
074haC3	Faecal	Rhi	0.00016	0.00004	0.00043	0.00027	**0.00025**
075pu1	Faecal	Pku	0.00016	0.00076	0.00200	0.00469	**0.00248**
075pu2	Faecal	Pku	0.00016	0.00054	0.00554	0.00276	**0.00295**
075pu3	Faecal	Pku	0.00016	0.00298	0.00113	0.00368	**0.00260**
0816ma1	Faecal	Ppi	0.00016	0.00007	0.00024	0.00044	**0.00025**
0816ma2	Faecal	Ppi	0.00016	0.00025	0.00002	0.00052	**0.00026**
0816ma3	Faecal	Ppi	0.00016	0.00017	0.00007	0.00058	**0.00028**
0913maD1	Faecal	Mda	0.00016	0.00006	0.00004	0.00028	**0.00013**
0913maD2	Faecal	Mda	0.00016	0.00013	0.00008	0.00055	**0.00026**
0913maD3	Faecal	Mda	0.00016	0.00027	0.00003	0.00038	**0.00023**
0927sa1	Faecal	Ppi	0.00016	0.00003	0.00005	0.00031	**0.00013**
0927sa2	Faecal	Ppi	0.00016	0.00001	-	0.00005	**0.00003**
0927sa3	Faecal	Ppi	0.00016	0.00001	0.00009	-	**0.00005**
074ha1	Faecal	Rhi	0.2	0.122	0.213	0.326	**0.22**
074ha2	Faecal	Rhi	0.2	0.150	0.049	0.349	**0.18**
074ha3	Faecal	Rhi	0.2	0.199	0.052	0.187	**0.15**
074ha1	Faecal	Rhi	0.04	0.012	0.036	0.065	**0.038**
074ha2	Faecal	Rhi	0.04	0.026	0.008	0.064	**0.033**
074ha1	Faecal	Rhi	0.008	0.0029	0.0061	0.0131	**0.007**
074ha2	Faecal	Rhi	0.008	0.0104	0.0042	0.0104	**0.008**
074ha3	Faecal	Rhi	0.008	0.0069	0.0018	0.0110	**0.007**
074ha1	Faecal	Rhi	0.000032	0.000007	0.000014	0.000092	**0.000038**
074ha2	Faecal	Rhi	0.000032	0.000016	0.000028	0.000066	**0.000037**
074ha3	Faecal	Rhi	0.000032	0.000032	0.000015	0.000034	**0.000027**

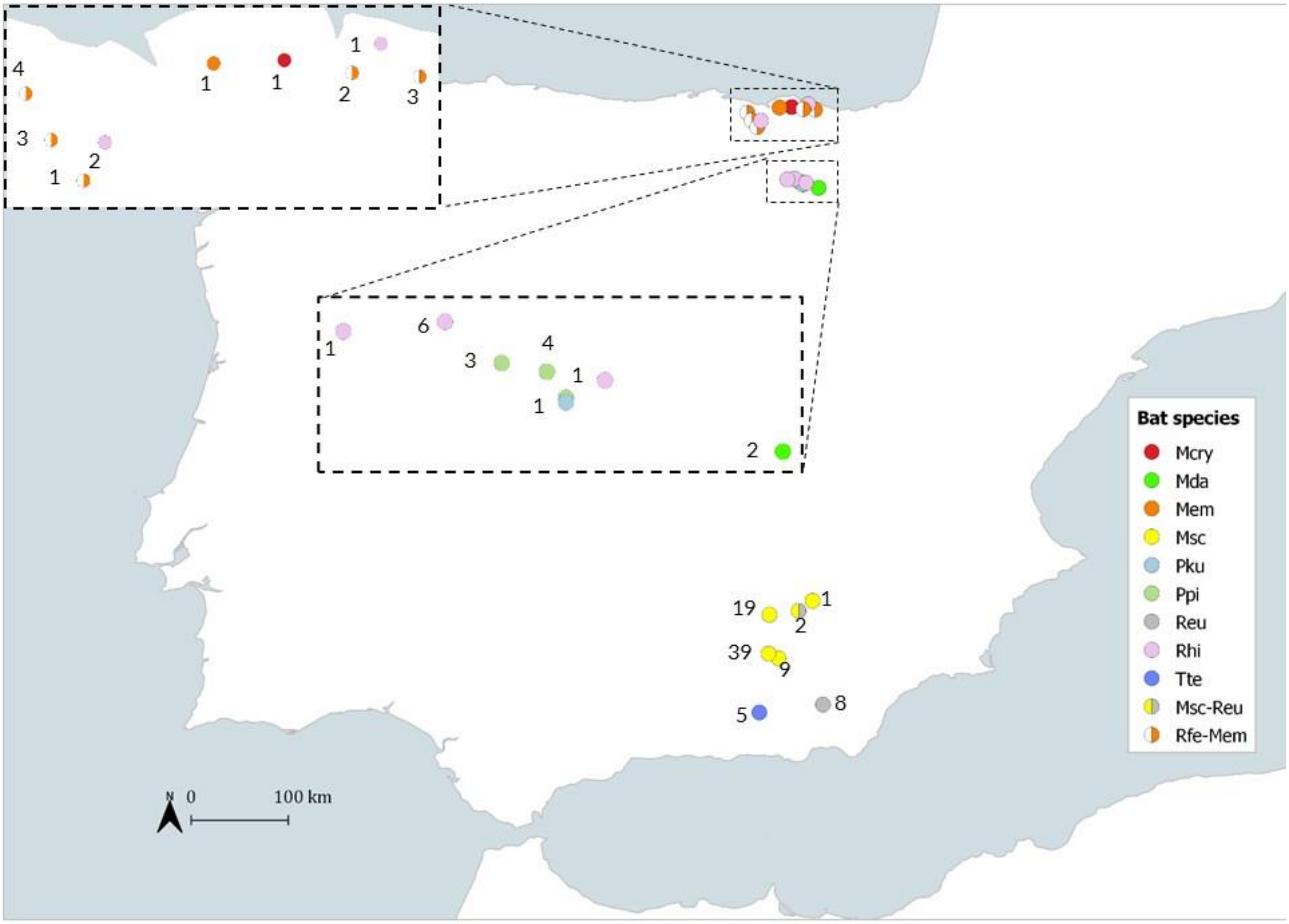

Figure S1. Geographic distribution of sampling locations and number of samples used for qualitative assessment. Mcry= *Myotis crypticus*; Mda= *M. daubentonii*; Mem= *M. emarginatus*; Msc= *Miniopterus schreibersii*; Pku= *Pipistrellus kuhlii*; Ppi= *P. pipistrellus*; Reu= *Rhinolophus euryale*; Rhi= *R. hipposideros*; Rfe= *R. ferrumequinum*; Tte= *Tadarida teniotis*.

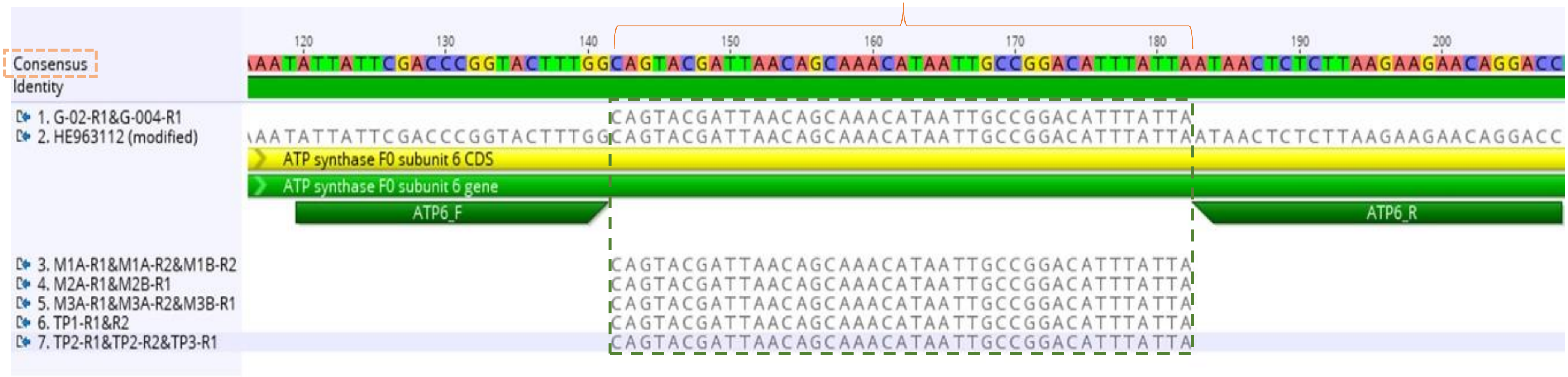

Figure S2. DNA sequencing results for the qPCR products: all amplicons corresponded to the same *T. pityocampa* sequence.
